# Why a Diffusing Single‐Molecule can be Detected in Few Minutes by a Large Capturing Bioelectronic Interface

**DOI:** 10.1002/advs.202104381

**Published:** 2022-05-06

**Authors:** Eleonora Macchia, Liberato De Caro, Fabrizio Torricelli, Cinzia Di Franco, Giuseppe Felice Mangiatordi, Gaetano Scamarcio, Luisa Torsi

**Affiliations:** ^1^ Faculty of Science and Engineering Åbo Akademi University Turku 20500 Finland; ^2^ CSGI (Centre for Colloid and Surface Science) Bari 70125 Italy; ^3^ Institute of Crystallography National Research Council via Amendola 122/O Bari 70126 Italy; ^4^ Dipartimento Ingegneria dell'Informazione Università degli Studi di Brescia via Branze 38 Brescia 25123 Italy; ^5^ Dipartimento di Chimica Università degli Studi di Bari “Aldo Moro,” Bari 70125 Italy; ^6^ CNR Istituto di Fotonica e Nanotecnologie Sede di Bari Bari 70125 Italy; ^7^ Dipartimento Interateneo di Fisica “M. Merlin,” Università degli Studi di Bari “Aldo Moro,” Bari 70125 Italy

**Keywords:** electrolyte‐gated field‐effect transistor, large‐capturing interface, organic bioelectronics, single‐molecule detection

## Abstract

Single‐molecule detection at a nanometric interface in a femtomolar solution, can take weeks as the encounter rate between the diffusing molecule to be detected and the transducing nanodevice is negligibly small. On the other hand, several experiments prove that macroscopic label‐free sensors based on field‐effect‐transistors, engaging micrometric or millimetric detecting interfaces are capable to assay a single‐molecule in a large volume within few minutes. The present work demonstrates why at least a single molecule out of a few diffusing in a 100 µL volume has a high probability to hit a large capturing and detecting electronic interface. To this end, sensing data, measured with an electrolyte‐gated FET whose gate is functionalized with 10^12^ capturing anti‐immunoglobulin G, are here provided along with a Brownian diffusion‐based modeling. The EG‐FET assays solutions down to some tens of zM in concentrations with volumes ranging from 25 µL to 1 mL in which the functionalized gates are incubated for times ranging from 30 s to 20 min. The high level of accordance between the experimental data and a model based on the Einstein's diffusion‐theory proves how the single‐molecule detection process at large‐capturing interfaces is controlled by Brownian diffusion and yet is highly probable and fast.

## Graphical abstract

A single‐molecule out of few in 100 µL diffusing according to Einstein's theory, can impinge on a large‐area (0.2 cm^2^) gate within 10 min. This is demonstrated by modeling the data gathered with a bioelectronic platform whose gate is functionalized with a highly packed layer of capturing‐antibodies. A general equation reproduces the data measured at different incubation volume and times.

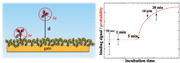

## 1 Introduction

The assay of biomarkers with a sensing system endowed with single‐molecule limit‐of‐detection (LOD),^[^ ^1^ ^]^ can be a game changer in enabling early detection of progressive diseases. For many years nanotechnologies seemed to offer feasible solutions by means of the so‐called near‐field approach at nanoscopic interfaces.^[^ ^2^ ^]^ However, the difference between single‐molecule resolution and single‐molecule LOD should be kept in mind. When a target molecule is dispersed in a volume of 10–100 µL comprising also the nanometric detecting interface, the interaction cross‐section between the two is extremely unlikely. This is known as the diffusion‐barrier issue,^[^ ^3^, ^4^ ^]^ which can be exemplified by saying that the probability for the interaction of a nanometric interface and a target molecule driven by diffusion is not negligible only within a volume of ≈1 µm^3^ (1 femtoliter, fL). Note that 1 particle in 1 fL corresponds to a nanomolar (nM) solution. As an instance, a simulation study shows that a timescale of several days are needed for ten molecules, out of 10^6^ in 100 µL, i.e., a femtomolar (fM) concentration, to hit a at a nanometric interface immersed in the same volume.^[^ ^4^ ^]^ Experimental evidences have been gathered on many nanotransducers,^[^ ^5^ ^]^ from nanopores ^[^ ^6^ ^]^ to nanotransistors,^[^ ^7^ ^]^ proving how they can detect with single‐molecule resolution, but only at LODs larger than picomolar (pM) concentration. Hence, nanointerfaces while being extremely effective to study rarer events offering indeed single‐molecule resolution but not low LOD, cannot be used to assay at extremely low concentration.

Large‐area or wide‐field ^[^ ^2^ ^]^ transducing interfaces, exposing a much larger active area can be a viable solution. However, it is often assumed that alike nanotransducing interfaces, also large‐area ones cannot detect a single molecule in a large‐volume (e.g., 10–100 µL), because affected by the diffusion‐barrier issue, too. This is proven erroneous by several published experimental pieces of evidence, involving mostly field‐effect transistor (FET) detections ^[^ ^8^ ^]^ at a large‐area interface. As an instance, a bioelectronic sensor based on a AlGaN/GaN high electron‐mobility transistor was proposed to detect antigen proteins such as Human Immunodeficiency Virus‐1 Reverse Transcriptase, Carcinoembryonic Antigen, N‐terminal pro b‐type natriuretic peptide, and C‐reactive protein (CRP), even in human serum at the fM concentration level.^[^ ^9^ ^]^ The gate surface, biofunctionalized with capturing antibodies, is 100 µm wide and the assay was completed in 5 min with the lowest detections in the fM range. Other highly performing bioelectronic sensors are the FETs gated via an ionically‐conducting and electronically‐insulating electrolyte, known as Electrolyte‐gated‐(EG)‐FETs,^[^ ^10^ ^]^ foreseen to be produced by scalable large‐area, low‐cost approaches.^[^ ^11^ ^]^ These sensors^[^ ^12^, ^13^ ^]^ are endowed with selectivity via the biofunctionalization of a millimeter‐wide sensing interface with a high density (up to 10^12^ cm^−2^) of recognition elements.^[^ ^14^, ^15^, ^16^ ^]^ An EG‐FET sensor with a graphene channel bearing 10^11^ cm^−2^ human olfactory receptors, can selectively bind the myl‐butyrate odorant marker down to a LOD of 40 attomolar (aM, 10^−18^ m) with a response‐time shorter than 1 s.^[^ ^17^ ^]^ Likewise, a graphene‐based EG‐FET was shown able to detect Anthrax Toxin at a LOD of 12 × 10^−18^ m, in 200 s.^[^ ^18^ ^]^ More recently the LOD was reduced down to tens of zeptomolar (zM) with the Single‐Molecule assay with a large‐Transistor (SiMoT) technology, involving an organic semiconductor based EG‐FETs.^[^ ^16^ ^]^ This is a single‐molecule assay as a 100 µL of a 10–20 × 10^−21^ m solution comprises 1 ± 1 molecules. Also in this case 10^12^ recognition elements were covalently attached at a millimeter‐wide gate (area of 0.2 cm^2^) electrode and detections were possible after 10 min of incubation in the solution to be assayed.^[^ ^19^ ^]^ Hence, SiMoT sets in 2018 a world record in label‐free single‐molecule detection and relevantly it was demonstrated to uniquely detect both proteins (CRP, IgG, IgM),^[^ ^16^, ^20^, ^21^, ^22^ ^]^ including virus capsids’ one (HIV‐p24),^[^ ^23^, ^24^ ^]^ aptamer,^[^ ^25^ ^]^ and genomic marker ^[^ ^26^ ^]^ also in serum. Lately, single‐molecule chiral ^[^ ^27^ ^]^ as well as CoV‐SERS‐2 virus ^[^ ^28^ ^]^ detection was proposed with large‐area, fast‐responding ad hoc functionalized organic FETs. The elicited pieces of evidence gathered on completely different FET structures by several research groups, demonstrate that a single molecule in 100 µL (concentration of ≈10–20 × 10^−21^ m) can diffuse and eventually impinge in the minute timescale, on a millimeter‐wide (e.g., ≈0.2 cm^2^) surface functionalized with trillions of recognition elements. The binding generates a signal that equals the noise average‐level plus three times its standard deviation (LOD definition).^[^ ^1^ ^]^ It is to point out that work is in progress to asses, if the response of a SiMoT device returns a reliable off/on type of response with a threshold at a LOD of a single marker in 100 µL, characterized by dynamic ranges that can vary from sample to sample.

The present work undertakes a systematic investigation to explain why when few molecules (< 10) diffuse in a large volume (e.g., 100 µL) comprising also a millimeter‐wide detecting interface, within 10 min at least one of them impinges on the large‐interface generating a detectable signal at the LOD. The engaged interface is the millimeter‐wide gate of a SiMoT EG‐FET device, covered by trillions of immunoglobulin G (anti‐IgG) capturing antibodies, while the target molecule is the IgG affinity antigen. The response data, acquired assaying solutions down to 60 ± 30 × 10^−21^ m encompassing different volumes (25 µL–1 mL) and incubation times (30 s–20 min), were successfully modeled with the Einstein's diffusion‐theory.

## 2 Results and Discussion

**Figure 1:**
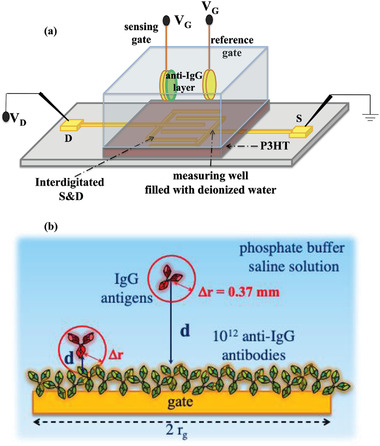
a) EG‐FET SiMoT set‐up comprising a P3HT channel coupled to either one of the two gates immersed in the well. b) Schematic cross‐sectional view of the incubation step carried out in the solution to be assayed (volumes of 100 µL or 1 mL) in contact with the functionalized sensing gate surface of radius *r*_g_. An IgG antigen can randomly move, in a time Δ*t*, within a sphere of radius Δ*r* being 0.37 mm in Δ*t* = 600 s.

In **Figure** **1**a, a typical EG‐FET SiMoT device structure is sketched. It includes a poly(3‐hexylthiophene‐2,5‐diyl) – P3HT FET channel with the source (S) and drain (D) interdigitated electrodes, along with a 5 mm diameter Au gate functionalized with a grafted layer of 10^12^ anti‐IgG capturing antibodies ^[^ ^16^ ^]^ addressed as sensing gate. A bare‐gold electrode, having the same size and serving as reference gate is also present. A well, glued around the channel, is filled with deionized water. This is addressed as the measuring well and the sensing and the reference gate immersed into it are, alternatively, capacitively coupled to the P3HT channel. The latter and the reference gate are always in the measuring well. The reference gate enables to control the level of the current flowing in the channel, at every stage of the sensing assay. Conversely, the sensing gate is alternatively immersed into the measuring well and into a separate one, addressed as incubation well, that contains the solutions to be assayed. The solutions to be assayed are based on phosphate buffered saline (PBS) so as to mimic a real fluid physiological high ionic strength of 162 × 10^−3^ m and pH of 7.4. They are spiked with a given number of IgGs. The necessity to separate the measuring from the incubation wells, is dictated by the need of measuring, while not screened, the electrostatic changes elicited by the IgG/anti‐IgG binding. Upon binding the sensing gate capacitively coupled to the FET channel undergoes a work‐function shift, measured as a change of the FET current. In deionized water, the Debye length is maximized and so is the FET current change.^[^ ^16^ ^]^

The incubation well is filled with IgG standard solutions, whose volume is 100 µL or 1 mL. A schematic diagram of the incubation process in these larger volumes is given in Figure 1b.

The sensing gate, whose radius (*r*_g_) is smaller than the edge of the incubation well (not shown), is depicted along with few IgG target antigens.

**Figure 2:**
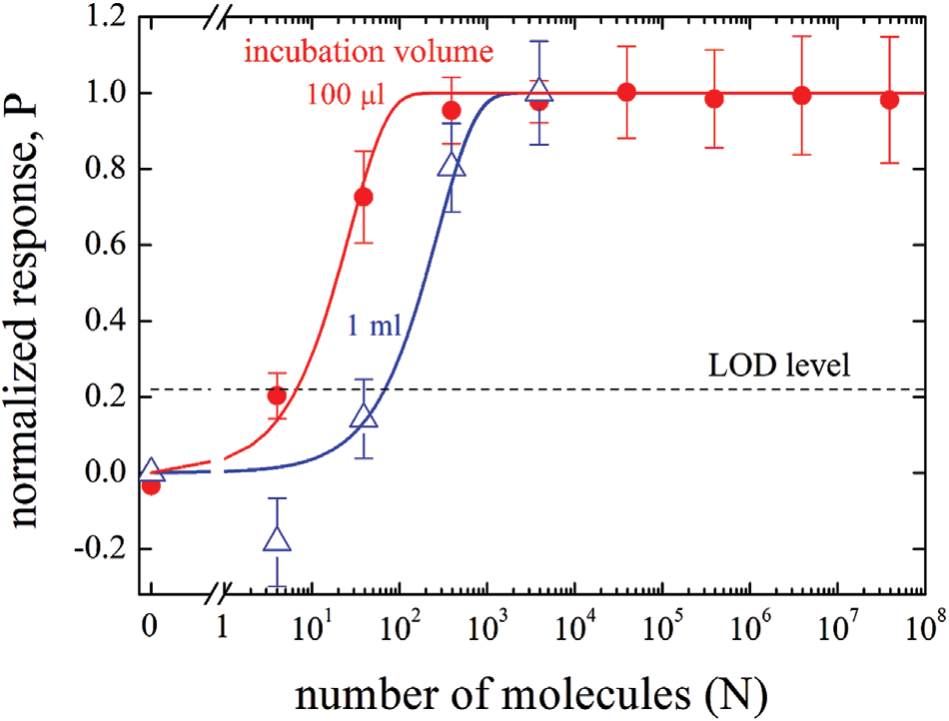
Normalized SiMoT EG‐FET responses, (Δ*I*/*I*_0_)/(Δ*I*/*I*_0_)^sat^ with (−Δ*I*/*I*_0_)^sat^ = 0.74 ± 0.13 for the data taken in 100 µL and (−Δ*I*/*I*_0_)^sat^ = 0.47 ± 0.14 for the data measured in 1 mL. The anti‐IgG functionalized gates were incubated for 10 min (600 s) into 100 µL (red symbols) and 1 mL (blue symbols) solutions of N IgGs, with N ranging from 4 ± 2 to 3.92 10^7^ ± 6 × 10^3^. Error bars are relevant to the reproducibility error indicated as one standard deviation over at least two replicates. On the *y*‐axis the P probability function (vide infra) is also given, and the solid lines are the result of the modelling. The black dotted line sets the level of the LOD.

In **Figure** **2**, the normalized electronic responses of the SiMoT EG‐FET devices are plot as a function of the total number of molecules, *N*, in the assayed volumes. The response is evaluated as the negative relative current shift upon sensing, namely (−Δ*I*/*I*_0_) = [−(*I* − *I*_0_)/I_0_], where *I*_0_ is the base‐line source–drain current measured after incubating the anti‐IgG biofunctionalized sensing gate in a PBS solution where no IgG molecules are present. The current *I* is measured after incubating the same gate in the solutions to be assayed encompassing a given number, *N*, of IgG molecules in each volume. The measured *I*–*V* transfer characteristics are given in Figure S1 and Section S1 (Supporting Information). The saturated value of (−Δ*I*/*I*_0_), (−Δ*I*/*I*_0_)_sat_ is sample dependent as it is related to the quantity of available anti‐IgG binding sites.^[^ ^21^ ^]^ As anticipated, the assayed samples are high ionic strength PBS standard solutions of the IgG antigens. The 100 µL of the PBS solution comprising 4 ± 2 IgG molecules where both the Poisson sampling and the concentration errors are evaluated at first.^[^ ^16^ ^]^ The same gate is incubated, afterward, in 100 µL solutions encompassing *N* = 39 ± 6, *N* = 392 ± 20, and *N* = 3.92 × 10^3^ ± 60, *N* = 3.92 10^4^ ± 2 × 10^2^, *N* = 3.92 × 10^5^ ± 6 × 10 ^2^, *N* = 3.92 × 10^6^ ± 2 × 10^3^, and *N* = 3.92 × 10^7^ ± 6 × 10^3^ IgG molecules, respectively. The data, given as red circles, are the average over at least two replicates (using two different anti‐IgG biofunctionalized gates), while the reproducibility error bars are given as one standard deviation. A similar dose curve is measured incubating anti‐IgG gates in a 1 mL (blue triangles) of PBS standard solutions. The minimum concentration assayed is 60 ± 30 × 10^−21^ m and the maximum is 600 ± 2 × 10^−15^ m. Relevantly, the encounter between the antigen and the capturing gate surface occurs during the incubation in the PBS standard solutions when no bias is applied so, no field induced drifting contributes to the antigen motion. The data for the incubation carried out in a smaller volume of 25 µL are given in Figure S2 and Section S2 (Supporting Information). In this case, a droplet was deposited on the gate as it was not possible to immerse the whole gate into such a small volume.

As customary for the SiMoT sensing protocol, the level of the source–drain current in the channel induced by a bare gold gate always kept in the measuring cell, is checked before and after the measurement of each dose curve, to control that its relative variation is within 5%.^[^ ^16^, ^19^, ^21^, ^22^, ^23^ ^]^ A negative control experiment was also performed by exposing an anti‐IgG functionalized gate to PBS standard solutions of the Immunoglobulin M (IgM) in the same concentration range span in the IgG assays. As IgM does not bind to anti‐IgG (Figure S3 and Section S3, Supporting Information), these data are taken as the noise, whose average level plus three‐times its standard deviation results in a LOD level of 22%, marking the lowest acceptable response at a confidence level of 99%.^[^ ^1^ ^]^

The solid curves in Figure 2 are calculated assuming a Brownian motion for the diffusion of the IgG molecules, undergoing stochastic collisions with the solvent molecules that are much smaller in mass and size.^[^ ^29^ ^]^ The Einstein's theory of diffusion relates the IgGs diffusion coefficient *D* to their mean squared displacement <Δ*r* ^2^> = <Δ*x* ^2^> + <Δ*y* ^2^> + <Δ*z* ^2^>, with

(1) $$\Delta r={\left(<\Delta r^{2}>\right)}^{1/2}={\left(6D\cdot \Delta t\right)}^{1/2}$$

*D* = *k*_B_ · *T*/(6 *π* · *η* · *a*), *k*_B_ is being the Boltzmann constant, *T* is the absolute temperature, *η* is the solvent viscosity, and *a* is the hydrodynamic radius of the diffusing Brownian protein. In Section S4 (Supporting Information) further details on the model are provided. The diffusion coefficient used, *D* = 3.89 × 10^−7^ cm^2^ s^−1^, is extracted from the analysis of photon‐correlation spectroscopy experiments for IgG monomers.^[^ ^30^ ^]^ Using the above expressions, it can be estimated that an IgG undergoing a Brownian motion for Δ*t* = 600 s (10 min) will span a spherical volume with a radius Δ*r* ≈0.037 cm. The portion of the solution that is close enough to the gate surface (*d* ≤ Δ*r*, see Figure 1b) to enable an antigen‐antibody interaction within 600 s can be approximated by a volume of *V*_Δ_ *_r_*, based on a cylindrical disk of radius *r*_g_ and height 0.5 · Δ*r*. The factor 0.5 accounts for the packing of a sphere of radius Δ*r* into a cube of edge 2 · Δ*r*. Hence, *V*_Δ_ *_r_* is given by

(2) $$V_{\Delta r}=\pi \cdot \Delta r\cdot 0.5\cdot {r^{2}}_{g}$$

One further aspect of the Brownian motion model worth commenting concerns the IgG rotational displacement angle for which equations and constrains equivalent to those given for the translational displacement hold. The time an IgG takes to span the whole solid angle 4*π* can be computed to be just 25 µs (Section S4, Supporting Information). Brownian motion, thus, also enables the antigen to quickly find the right orientation to correctly bind a given antibody, even when the latter is not perfectly oriented toward the solution to be assayed.

The fraction *f* of the incubating volume *V* that is close enough to the gate surface to enable the antigen‐antibody interaction within 600 s, is

(3) $$f=\left(V_{\Delta r}/V\right)=\left(\pi \cdot 0.5\cdot \Delta r\cdot {r^{2}}_{g}\right)\cdot /V$$

Plugging Equations (1) into (3) the following results

(4) $$f=\left[\frac{\pi }{2}\sqrt{6D\Delta t}\cdot r_{g}^{2}/V\right]$$

The SiMoT EG‐FET response can be elicited by just one antigen being captured.^[^ ^11^, ^16^, ^21^ ^]^ To model this occurrence, assuming that *N* IgG antigens are randomly dispersed in the assayed volume *V*, *f* is defined as the probability that one of them happens to be sufficiently close to the gate surface (namely, within a Δ*r* distance), to eventually collide on it. The (1 *– f*) term is the probability that no antigen is in the *f* portion of the volume closer to the gate surface. Hence, the conditional probability that at least one antigen, out of *N*, is in the proximity by the gate surface, is given by

(5) $$P=f+\left(1-f\right)\cdot f+{\left(1-f\right)}^{2}\cdot f+\cdot \cdot \cdot {\left(1-f\right)}^{N-1}\cdot f$$

The rationale behind Equation (5) is to model a system of *N* diffusing IgGs by the conditional probability function *P* built as follows: one IgG out of the *N* present in the assayed volume *V*, holds a probability *f* to find itself in the *V*_Δ_ *_r_* fraction of the whole volume (Equation (3)). This IgG will be, hence, close enough to the gate to hit against the anti‐IgG functionalized surface (Figure 1b) within a Δ*t* of 10 min. At this point the binding can easily take place as the IgG spans, rotating, the whole solid angle in only 25 µs. Thus, the antigen can quickly find the right orientation to bind the anti‐IgG bumped into, out of the 10^12^ antibodies populating the gate surface. According to the hypothesis, a second IgG can be wherever, but in the volume *V*_Δ_ *_r_*. So, the second term in Equation (5), (1 *– f*) · *f*, expresses the probability that no second IgG is in the *f* portion of the volume closer to the gate surface. A third IgG will also not be in the *f* fraction and the third term will be (1 *– f*) ^2^· *f*; and so on for the other N‐4 particles populating the volume.

Relevantly Equation (5) encompasses only independently measured physical parameters such as the Δ*t* incubation time, the volume *V* assayed, the diffusing Brownian coefficient *D* for an IgG monomer.^[^ ^30^ ^]^ The probability *P* is given as a function of the incubation volume encompassing different *N* values in Figure S4 and Section S5 (Supporting Information). The complete expression of Equation (5) is provided in Section S5 (Supporting Information) along with the list of the physical quantities and constants used for the calculations (Table S1, Supporting Information).

The plot of the *P* probability function (Equation (5)) results in the solid curves given in Figure 2. Indeed, within one standard deviation all the experimental trends, are reproduced by the model. The very good agreement between the experimental data reported in Figure 2 and Equation (5) is further proven by the chi‐squared test (Section S6, Supporting Information) assessing a very high chance of 95% that such experimental data are successfully predicted by the P’ modeling function. The modeling curves serves also to assess the number of molecules at the LOD level, *N*_LOD_, being 8 ± 3 for the 100 µL data and 175 ± 13 the 1 mL ones.

To deepen the understanding of the phenomenon investigated, the SiMBiT EG‐FET Δ*I*/*I*_0_ responses measured at different incubation times, namely Δ*t* of 30 s, 1, 5, 10, and 20 min, are modeled. To this end, the so far used anti‐IgG functionalized gates are engaged to assay a 100 µL solution of *N* = 39 ± 6 IgG molecules.

**Figure 3:**
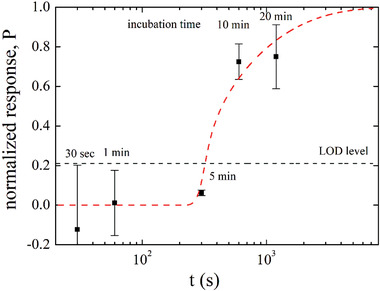
Normalized SiMoT EG‐FET responses (Δ*I*/*I*_0_)/(Δ*I*/*I*_0_)^sat^ with (−Δ*I*/*I*_0_)^sat^ = 0.74 ± 0.13. Anti‐IgG functionalized gates incubated for Δ*t* 30 s, 1, 5, 10, and 20 min into a 100 µL volume containing a 39 ± 6 × 10^−21^ m IgGs. Error bars are the reproducibility standard deviations over two replicates. On the *y*‐axis the P probability function is also given, and a solid red line is the result of the modeling; the dotted portion of the red line is relevant to the timeframe (Δ*t* < 250 s) in which the P functions does not return a physically meaningful value. The black dotted line sets the level of the LOD.

The data are plotted in **Figure** **3** as the normalized Δ*I*/*I*_0_ versus the incubation time Δ*t* along with the *y*‐axis error bars taken as one standard deviation over two replicates. In this case, 10 different solutions are assayed with an equal number of different gates. As it is apparent an incubation time Δ*t* between 5 and 10 min is needed to see a response beyond the LOD level. The modeling is carried out considering the very same function P given by Equation (5), that can indeed model the (−Δ*I*/*I*_0_) response as a function of the incubation time Δ*t*, because it implicitly depends on the square‐root of the time via the Stokes–Einstein relation (Equation (1)). The *P* probability function (red line in Figure 3) reproduces very well the experimental data as assessed, also in this case, by the chi‐squared test (Section S6, Supporting Information). Equation (5) returns no response for Δ*t* < 250 s (4.17 min, dotted portion of the red line) as this is a too short timeframe for at least one IgG out of 39 to impinge at the gate in 100 µL. The ability of Equation (5) to model also the responses as a function of the incubation time confirms the validity of a model based on the Brownian motion here discussed and confirms that with an incubation time of 9.2 min at least one‐single molecule binding gives origin to a signal at the LOD level. This clearly proves that in 100 µL out of 8 ± 3 IgG molecules (*N*_LOD_ in 100 µL) and in only in 9.2 min (Δ*t*_LOD_) at least one IgG reaches by pure diffusion the millimeter‐wide gate. This demonstrates that the diffusion barrier issue does not apply when a millimeter wide interface is used to assay a volume of 100 µL or 1 mL, so that a SiMoT device can sense down to a LOD of 130 × 10^−21^ m or lower depending on the measured level of the LOD. As an instance with a LOD level of 11.6%,^[^ ^16^ ^]^ a LOD of 68 × 10^−21^ m could have been reached. This also implies that a single IgG protein (footprint ≈10^2^ nm^2^ = 10^−12^ cm^2^) can be successfully detected at the 0.2 cm^2^ gate detecting surface, despite the 10^11^ orders of magnitude difference. Indeed, it is an ingrained belief that, the negligible footprint of a single molecule onto a many orders of magnitudes larger interface, returns a weak signal falling under the noise level. Experimental data prove this statement wrong. As an instance, some cells,^[^ ^31^, ^32^, ^33^ ^]^ can detect a single photon, or a single chemoattractant, via their surface packed with capturing recognition elements. Indeed, amplification effects must be in place because a footprint of a single molecule (nanometric in size) is at least 10^8^ times smaller than the area of a cell surface (0.01–0.10 mm in size). Such occurrences, that are seen both in cells and FET‐biosensors necessarily call for amplification effects discussed elsewhere.^[^ ^16^, ^19^, ^21^ ^]^

## 3 Conclusion

FET bioelectronic sensors comprising a millimeter‐wide detecting gate covered by trillions of capturing elements such as antibodies, were proven capable to detect proteins at ultralow concentrations in the timeframe of minutes by several groups working on different technologies. Particularly relevant in this scenario is the SiMoT technology that has enabled single‐molecule detection of proteins and genomic markers at the tens of zeptomolar concentration level, after an incubation time of only 10 min. These bioelectronic sensors can be fabricated by scalable large‐area and cost‐effective technologies and require no pretreatment or preparation of the sample to be assayed. Hence, they hold a tremendous potential in ultimately sensitive and fast detection of markers or even pathogens.

The sensing mechanism of this innovative approach is still under scrutiny and the present work adds a critically important piece of information: a single‐molecule out of few, acting as a Browning particle diffusing according to the Einstein's diffusion‐theory, can impinge on a large‐area gate, functionalized with trillions recognition elements, within 10 min. This is demonstrated by modeling the experimental data gathered with EG‐FET SiMoT devices with a very simple expression (Equation (5)) based on the Brownian theory. The acquired data, involves both the sensor response measured as a function of the volumes assayed at different concentrations (dose curves) and as a function of the incubation time. The data are very well reproduced by the same equation. Relevantly, the model also reveals that the fast spinning of the diffusing antigen enables also to quickly find the right orientation to bind to one capturing element independently of its orientation.

This work demonstrates that the diffusion‐barrier issue, impairing the use of a single‐molecule detection at a nanometric interface to assay solution with concentrations below picomolar, does not apply when the same experiment is conducted with a FET bioelectronic sensor comprising a micrometric or a millimetric wide detecting interface. It this case a single molecule can be detected within few minutes in a 100 µL solution with a concentration down to few tens of zeptomolar.

## 4 Experimental Section

### Materials

The organic semiconductor channel material is a poly(3‐hexylthiophene‐2,5‐diyl), P3HT (regioregularity > 99%), with an average molecular weight of 17.5 kDa (g mol^−1^), used with no further purification. 3‐mercaptopropionic acid (3‐MPA) and 11‐mercaptoundecanoic acid (11‐MUA), 1‐ethyl‐3‐(3‐dimethylaminopropyl)‐carbodiimide (EDC), *N*‐hydroxysulfosuccinimide sodium salt (sulfo‐NHS), and K4[Fe CN)6]·3H2O (98.5%) purchased from Sigma‐Aldrich were also used with no further purification. The anti‐Human Immunoglobulin G (anti‐IgG, Sigma‐Aldrich Product No. I2136) is a polyclonal antibody (molecular weight ≈144 kDa), while the human IgG (≈150 kDa) affinity ligand were extracted from human serum. Purified human IgG, purchased from Sigma‐Aldrich (Product No. I 2511), is produced by precipitation and gel filtration techniques using normal human serum from one healthy donor as the starting material, to prevent the presence of dimer fraction in the sampled solution. The source material has been tested and found negative for antibody to HIV, antibody to HCV and for HbsAg. Bovine serum albumin (BSA) has a molecular weight of 66 kDa. All the proteins were purchased from Sigma‐Aldrich and readily used. Water (HPLC‐grade, Sigma‐Aldrich), potassium chloride (Fluka, puriss p.a.) and ethanol grade, puriss. p.a. assay, ≥ 99.8%, were also used with no further purification. All the electrical characterization and sensing experiments have been performed by means of a Keithley 4200‐SCS semiconductor characterization system in air at room temperature in a dark box. All data were treated using OriginPro 2018, while Brownian diffusion‐based modeling was implemented by means of Wolfram Mathematica software.

### Preparation of the IgG Standard Solutions

The IgG PBS solutions were prepared by a serial dilution process with the dilution factor given by: *c*_1_ *x V*_1_ *= c*_2_ *x V*_2_, where *c*_1_ and *c*_2_ are the ligand concentrations in stock and in the diluted solution respectively, while *V*_1_ and *V*_2_ are the volumes of the stock and of the diluted solution, too. As customary, in a serial dilution process the former dilution is the stock solution for subsequent dilution in the series. The nominal number of the IgG proteins (# IgG) at each concentration was estimated as $\#IgG=\frac{c}{VN_{A}}$, where *c* is the ligand concentration, *V* is the volume of the standard PBS solution in which the gate is incubated, ranging from 25 µL to 1 mL, and *N*_A_ is the Avogadro number. The uncertainty associated with the sampling in the serial dilution can be estimated, according to the Poisson's distribution, as the square root of the expected number of IgG proteins corresponding to one standard deviation.

### SiMoT Electrolyte Gated FET Fabrication

The EG‐FETs, schematically shown in Figure 1a, were fabricated on a silicon substrate, covered by a 300 nm thick SiO_2_ layer. Source (S) and drain (D) interdigitated electrodes were photolithographically defined on the substrate and covered by a thiophene‐based organic semiconductor ^[^ ^34^ ^]^ Electron‐beam evaporated Au films (50 nm thick) were deposited on an adhesion layer of Ti (5 nm thick). The channel length, 5 µm, and the channel width, 10.560 µm, define an effective channel area of 5.3 × 10^−2^ mm^2^. A solution of P3HT (2.6 mg mL^−1^ in chlorobenzene, filtered through a 0.2 µm sieve) was spin‐coated at 2 × 10^3^ r.p.m. for 20 s on these electrodes and annealed at 90 °C for 15 s. A polydimethylsiloxane well was glued around the interdigitated channel area and filled with 300 µL of deionized water (HPLC‐grade) serving as gating medium.^[^ ^35^ ^]^ This is the SiMoT EG‐FET measuring well comprising also two gate (G) electrodes. They hold a circular area of ≈0.2 cm^2^ and a *r*_g_ = 0.25 mm and were fabricated on PEN foil substrates by shadow‐mask lithography and e‐beam evaporation of Ti/Au (5/50 nm) films. The gate area being ≈10 times that of the channel assures that the current relative change is mostly due to the shift of the gate work‐function, or equivalently, the threshold voltage.^[^ ^16^ ^]^ The gate serving as sensing gate undergoes a biofunctionalization process (vide infra) to be covered by the capturing anti‐IgG antibodies. The other gate, addressed as reference gate, is made of bare‐gold and measures the current level in the FET channel at any stage of a sensing measurement.

### Gate Biofunctionalization Protocol

The sensing gate electrode was biofunctionalized according to a protocol described elsewhere.^[^ ^16^, ^21^ ^]^ It comprises a 3‐MPA and 11‐MUA mixed chemical self‐assembled monolayer (SAM) activated with EDC‐sulfoNHS chemistry to whom anti‐IgG capturing proteins are covalently attached. The unreacted activated carboxylic groups are deactivated in ethanolamine. The protocol enables to reach a coverage of capturing anti‐IgG of 6 ×10^11^. BSA is also physisorbed to minimize nonspecific binding. The binding properties of the IgG analyte to the anti‐IgG capturing layer is independently assessed by mean of a surface plasmon resonance characterization (Figure S5 and Section S7, Supporting Information).

### Sensing Measurements

The reference bare gold gate is always in the measuring well and enables to control the level of the current flowing in the P3HT channel at every stage of the sensing assay. It is relevant to point out that the need for an Ag/AgCl reference electrode is not strictly required to control potential in a solution in the absence of faradaic currents.^[^ ^19^ ^]^ On the other hand, the integration of the Ag/AgCl electrodes in a circuit is still a major technological issue, therefore it was decided to avoid its use. The EG‐FET with a gold electrode takes some hours to stabilize but this is a process that is needed only after fabricating the device. Afterward, the device is very stable for days.^[^ ^36^ ^]^ Hence, this is very acceptable and makes the SiMBiT system much more technologically appealing. Relevantly, the presence of the gold electrode in the cell does not affect at all the operation of the sensor.

The biofunctionalized sensing gate goes from the incubation and the measuring well and it was proven that this does not provoke a shift the measured FET current of more than few %.^[^ ^16^ ^]^ Before proceeding with the sensing measurements, the source–drain FET current is stabilized recording subsequent repeated measurements of the EG‐FET *I*–*V* transfer curve (*I* vs the gate bias at a fixed source–drain bias of −0.3 V) in the measuring well using the reference gold gate, until a stable current is measured for three subsequent cycles. The sensing gate is then incubated in a separate well addressed as incubation well (or it is exposed to a droplet of the solution to be assayed) of a given volume of phosphate‐buffered saline (PBS, ionic strength of 162 × 10^−3^ m and pH of 7.4) solution (at RT and in the dark) for 10 min. The gate is used directly after the incubation stage and washing (vide infra) and the stabilization of the gate after incubation and washing takes 5–10 min. Afterward the sensing gate is washed thoroughly with HPLC water to remove the excess of salts and/or antigen stuck on the surface. It is then transferred in the EG‐FET measuring well and a new cycle of transfer characteristics is registered.^[^ ^16^ ^]^ Upon measurement of a stable *I*_0_ base line, the same sensing gate is removed from the measuring well and transferred back into the incubation well filled with a PBS standard‐solutions of the IgG molecules dispersed in different volumes. The incubations are caried out also for different given timeframes. Specifically, after incubation in each of the PBS standard‐solutions the gate is washed thoroughly with HPLC grade water to remove physisorbed proteins, and the *I*–*V* transfer curve is measured in the measuring well.^[^ ^16^ ^]^ The stabilized currents measured after incubation in each standard solution are addressed as the “I” signal at a given concentration. The (−Δ*I*/*I*_0_) = −(*I* − *I*_0_)/*I*_0_ is the electronic response at a given volume/incubation time and the relevant curves are obtained by plotting the data at the gate‐bias value that maximizes the trans‐conductance *δI*/*δV* (falling generally in the −0.3 to −0.4 V range), at all the investigated volumes/incubation‐times. All the data points are averaged over two or three replicates and the reproducibility error is computed as one standard deviation.

## Conflict of Interest

The authors declare no conflict of interest.

## Supporting information

Supporting Information

## Data Availability

The data that support the findings of this study are openly available in FairData at https://ida.fairdata.fi/s/NOT_FOR_PUBLICATION_si6dWSDE3NLN, reference number 2004602.
