# Supplementary material for: Why a Diffusing Single‐Molecule can be Detected in Few Minutes by a Large Capturing Bioelectronic Interface
Source: Adv Sci (Weinh). 2022 May 6;9(20):2104381. doi: 10.1002/advs.202104381 (PMC9284160; doi:10.1002/advs.202104381)
Supplement: Supplementary file 1 — Supporting Information [file ADVS-9-2104381-s001.pdf]

ADVANCED SCIENCE
Open Access

Supporting Information

for *Adv. Sci.*, DOI 10.1002/advs.202104381

Why a Diffusing Single-Molecule can be Detected in Few Minutes by a Large Capturing Bioelectronic Interface

Eleonora Macchia, Liberato De Caro, Fabrizio Torricelli, Cinzia Di Franco, Giuseppe Felice Mangiatordi, Gaetano Scamarcio and Luisa Torsi**

Supporting Information

Why a diffusing single-molecule can be detected in few minutes by a large capturing bioelectronic interface

Eleonora Macchia, Liberato De Caro, Fabrizio Torricelli, Cinzia Di Franco, Giuseppe Felice Mangiatordi, Gaetano Scamarcio and Luisa Torsi**

Section 1: SiMoT I-V transfer characteristics

The drain current (I_D) – gate voltage (V_G) transfer curves at a fixed drain voltage V_D = - 0.4 V are shown in **Figure S1a**. The curves were measured in the forward and reverse mode to evidence the occurrence of any hysteresis. The black curve is the current measured on a sensing gate biofunctionalized with anti-IgG incubated for 10 minutes in 100 μL of bare PBS after a stable current level has been accomplished. The measurement was carried out by sweeping the gate bias from 0.1 V to - 0.5 V with steps of 10 mV in water. The black curve has been taken as the *baseline*. Subsequently, the very same sensing gate has been incubated for 10 minutes in 100 μL of PBS standard solutions comprising 4 ± 2 IgG molecules, and the red curve has been measured. The same gate has been afterwards incubated in 100 μL solutions encompassing N = 39 ± 6, N = 392 ± 20, N = 3.92 10^3 ± 60, N = 3.92 10^4 ± 2 10^2, N = 3.92 10^5 ± 6 10^2 IgG molecules. Remarkably a significant current decrease has been registered already upon exposure to 4 ± 2 IgG molecules. This trend is replicated as the standard solutions with increased IgG concentration are progressively assayed, until the saturation of the response is reached.

The curves given in **Figure S1b** are the correspondent gate leakage currents I_G, which are always about three orders of magnitude lower than I_D. No faradaic activity can be evidenced by the I_G curve of the anti-IgG gate, proving that mild electrochemical processes have been prevented by the fine-tuning of the inspected gate voltage window. Moreover, **Figure S1b** shows also no correlation between the I_G current and the ligand concentration.

These are compelling evidence, proving that the field-effect induced current I_D provides a capacity-coupled related sensing response. No other current flowing in the device can provide the same information.

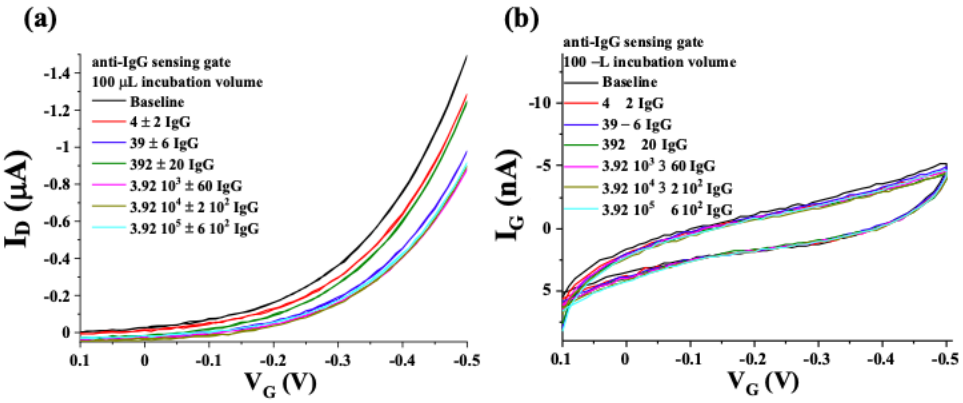

Figure S1. ***(a)*** *Transfer characteristics,* I_D *(drain current) -* V_G *(gate bias) at* V_D *(drain bias) = - 0.4 V,* ***(b)*** *along with the* I_G *(gate current) -* V_G *curve at* V_D *= - 0.4 V, registered upon exposure to PBS, being the baseline (black curve) and to 100 μL of PBS standard solution of IgG comprising* 4 ± 2 *(red curve),* 39 ± 6 *(blue curve),* 392 ± 20 *(green curve),* $3.92\ 10^3 \pm 60$ *(magenta curve),* $3.92\ 10^4 \pm 2\ 10^2$ *(yellow curve) and* $3.92\ 10^5 \pm 6\ 10^2$ *(cyan curve) molecules, progressively assessed with the same anti-IgG biofunctionalized sensing gate. The gate is incubated in the samples to be assayed for 10 minutes. Here 20 transfer characteristics are measured. In the panel, only the curves measured during the last (20th) cycle at each concentration assayed are shown.*

Section 2: The 25 μl incubation volume case

For the smallest volume assayed, a 25 μl, a droplet is deposited on the sensing gate. A schematic of the smallest volume assayed with the 25 μl droplet deposited directly on the gate surface, is also provided (**Figure S2a**). In this case, the radius of the droplet r_d is measured to be 0.34 cm while that of the gate is r_g = 0.25 cm as shown in **Figure S2b**. **Equation 2** in the main text holds also in the case of the sensing gate exposed for 10 minutes to 25 μl droplet of the PBS standard solutions. **Figure S2a** shows that the whole gate area is covered by the droplet while the incubation system is schematically depicted in **Figure S2b.** The volume of a liquid droplet with contact angle θ and droplet radius r_d is $V_d = \pi \cdot r_d^3 \cdot (2 - 3\cos\theta + \cos^3\theta)$ /

$(3 \cdot \sin^3\theta)$.[1] For $r_d \sim 3$ mm a contact angle θ of about 60° is computed, accounting for the relatively high wettability of the densely packed anti-IgG surface.

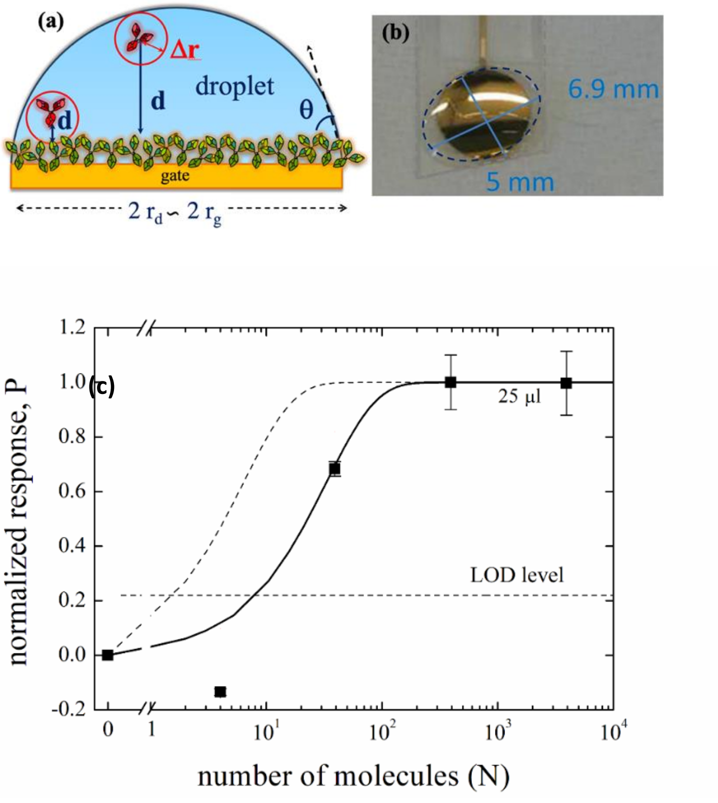

***Figure S2** – (**a**) Schematic cross-sectional view of the incubation step carried out in a droplet (volume = 25 µl) of radius r_d, forming a contact angle $\theta \sim 60°$ with the gate surface. **(b)** Top-view picture of the sensing gate covered by a 25 µl droplet during the incubation. **(c)** Normalized SiMoT EG-FET responses, $(\Delta I/I_0)/(\Delta I/I_0)^{sat}$ with $(-\Delta I/I_0)^{sat} = 0.65 \pm 0.12$, of anti-IgG functionalized gates incubated for 10 minutes (600 s) into a solution of IgG of 25 µl (black symbols) with the N number of IgG ligands ranging from 4 ± 2 to $3.92\ 10^4 \pm 2 \cdot 10^2$. Error bars are relevant to the reproducibility error indicated as one standard deviation over at least two replicates. On the y-axis the P probability function is also given, and the solid lines are the result of the modelling with the P function where the f function (Equation 3, main text) is multiplied by a factor ~ 0.19. The dotted line is the results of the modelling with the P probability function. The dotted horizontal line is the level of the LOD.*

This is in line with a measured contact angle of 65° reported for a surface covered with a protein density of 0.25 µg/cm^2,[2] comparable to the 10^{12}/cm^2 anti-IgGs covering the SiMoT gate surface. Also in this case, however, the portion of the solution that is close enough to the

gate surface ($d \leq \Delta r$, see **Figure 2c**) to enable an antigen-antibody interaction within 600 s, can be approximated by a volume $V_{\Delta r}$ given by **Equation 2**, as $\theta \sim 90°$ and the cylindrical disk has a radius r_d with a height of Δr.

The plot of the *P* probability function **(Equation 5)** results in the dotted curve given in **Figure S2c**. As it is apparent the P function is unable to reproduce the 25 μl data that are shifted towards higher N values. To fit the actual data the *f* function defined in the main text by the **Equation 3** (main text) needs to be multiplied by a factor of ~ 0.19. Assuming a constant Δ_R these results suggest a reduced effective surface in the case of the incubation by drop casting. Qualitatively, these findings may be explained by the well-known spontaneous dragging of liquid from the droplet's interior towards its edges (so called coffee-ring effect)[3],[4] that may preferentially distribute the diluted IgG molecules in the outer part of the droplet and reduce the effective interaction area of the gate.

Section 3: Evaluation of the limit-of detection (LOD)

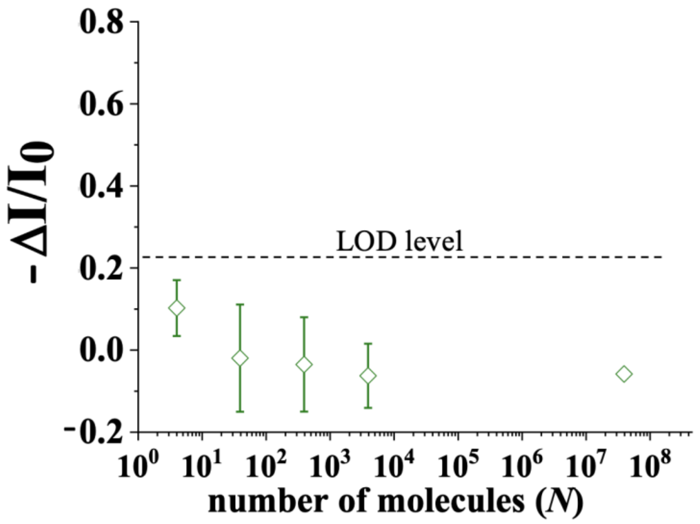

Figure S3 *- Response of anti-IgG functionalized gates exposed to solutions of IgM molecules in PBS. Error bars are standard deviations over three replicates. The LOD level is computed as the average level of the signal (noise) plus three times its standard deviation.*

The negative control experiment performed by exposing an anti-IgG gate to PBS standard solutions of Immunoglobulin M (IgM), defining the noise, is given in **Figure S2.**

Section 4: Further details on the Brownian motion theory

The translational diffusion coefficient D is given by the Stokes-Einstein equation:

$$D = k_B \cdot T / (6 \pi \cdot \eta \cdot a) \tag{S3.1}$$

with k_B being the Boltzmann constant, T the absolute temperature, η the solvent viscosity and *a* the hydrodynamic radius of the diffusing Brownian protein. Given the solute concentration c = 10 mM - KCl 2.7 mM and 137 mM NaCl - in the standard PBS solution (PH 7.4 and ionic-strength of 162 mM), we obtain a viscosity η_s:[5]

$$\eta_s/\eta = 1+A\ c^{1/2}+B\ c \tag{S3.2}$$

where $\eta = 1\cdot10^{-2}$ $g\cdot cm^{-1}\cdot s^{-1}$ is the water viscosity at room temperature. In **Equation S3.2** $A/(mol/l)^{-1/2}$ and $B/(mol/l)^{-1}$ are equal to 0.0062 and 0.0793 for NaCl; these constants are equal to 0.0052 and -0.0140 for KCl. Inserting these values and the solute concentrations of the two salts in **Equation S3.2**, we obtain $\eta_s = 1.013 \cdot 10^{-2}$ $g\cdot cm^{-1}\cdot s^{-1}$, very close to the value for water. Taking *a* = 5.51 ± 0.03 nm, the value for IgG monomers,[6] we obtain from Equation S1 a value D = 3.92 ± $0.02\cdot10^{-7}$ cm^2/s, in full agreement with photon-correlation spectroscopy data (3.89 ± $0.02\cdot10^{-7}$ cm^2/s).[6]

The dynamics of an IgG moving in a PBS (water) solution is described by the Langevin equation adapted to the case of an antigen - antibody interaction,[7] with the trajectory generated as a set of snapshots of the antigen position at each Δt time interval. The force term in the Langevin equation encompasses only the random walk (Brownian motion effect), assuming a negligible effect of hydrodynamic and gravitational forces as well as electrostatic interactions. The latter was chosen based on the results of the SiMoT assays involving many proteins, DNA strands and we have seen both current decreases and current increases. For

instance, in the assay of IgG, IgM and many others the current decreases while with peptides and bacteria (private communication) we see a current increase. It is a fact that we can detect, invariably, all these in 10 minutes. So, under the described experimental conditions the electrostatic interaction does not play a crucial role.

The translational displacement Δ**r** is given by: $\Delta\mathbf{r} = (k_B\,T)^{-1} \cdot D \cdot \mathbf{F} \cdot \Delta t + \mathbf{R}$ where **F** is the force acting on the antigen before the step is taken, **R** is the random vector satisfying both $\langle \mathbf{R} \rangle = 0$ and $\langle \mathbf{R}^2 \rangle = 6\, D \cdot \Delta t$. The factor $[(k_B\,T)^{-1} \cdot D]$ models the dumping effect generated by the friction with the molecules of the solvent. As in the case under study, the anti-IgG capturing antibodies are covalently attached at the gate surface while the IgG antigen diffuses in the solvent, the latter can be treated as a rigid body whose translational motion is referred to the position of the gate. For the diffusion to proceed it is necessary that, within Δt, the forces and the torques acting on the IgG antigen, as well as the gradient of any diffusion tensor, remain effectively constant. Hence, the motion should be considered only over timeframes exceeding the momentum relaxation time $\Delta t_r = (m \cdot D) / k_B\, T$ with m being the antigen mass. Plugging $m \sim (\rho \cdot 4\pi \cdot a^3) / 3$ and **Equation S3.1** into Δt_r, results in:

$$\Delta t_r = 2a^2 \cdot \rho / (9 \cdot \eta) \qquad \text{(S3.3)}$$

that falls in the picosecond range. This proves that the condition for the Brownian motion to hold, is met. It is straightforward to show that hydrodynamic interactions and gravitational effects can be neglected.

The Brownian motion give rise also to a rotational diffusion with a random rotational angle **W** satisfying $\langle \mathbf{W} \rangle = 0$ so that

$$\langle \mathbf{W}^2 \rangle = 6\, D_R\, \Delta t. \qquad \text{(S3.4).}$$

with a D_R, rotational diffusion coefficient being, according to the Einstein–Smoluchowski $D_R = k_bT/8\pi\eta a^3$. For an antibodies $D_R \sim 1$ MHz. If we put $(\langle \mathbf{W}^2 \rangle)^{1/2} = 4\pi$, $a = 5.51$ nm, from

Equation 2.4 we obtain that the time an IgG takes to span the whole solid angle 4π, is $\Delta t_{4\pi}=16\pi^2/6D_R \sim 25\ \mu s$.

Section 5: Complete expression of the P probability function

The complete expression of the probability function P is provided in the following **Equation S4.1**:

$$P = \left[\frac{\pi}{2}\sqrt{6D\,\Delta t}\ \cdot r_g^2/V\right]\left\{1+\left[1-\frac{\pi}{2}\sqrt{6D\,\Delta t}\ \cdot r_g^2/V\right]+\left[1-\frac{\pi}{2}\sqrt{6D\,\Delta t}\ \cdot r_g^2/V\right]^2+\cdots\left[1-\frac{\pi}{2}\sqrt{6D\,\Delta t}\ \cdot r_g^2/V\right]^{N-1}\right\} \quad \text{(S4.1)}$$

Details on the probability function P as function of the incubation volume encompassing a different number of molecules N is given in **Figure S4**.

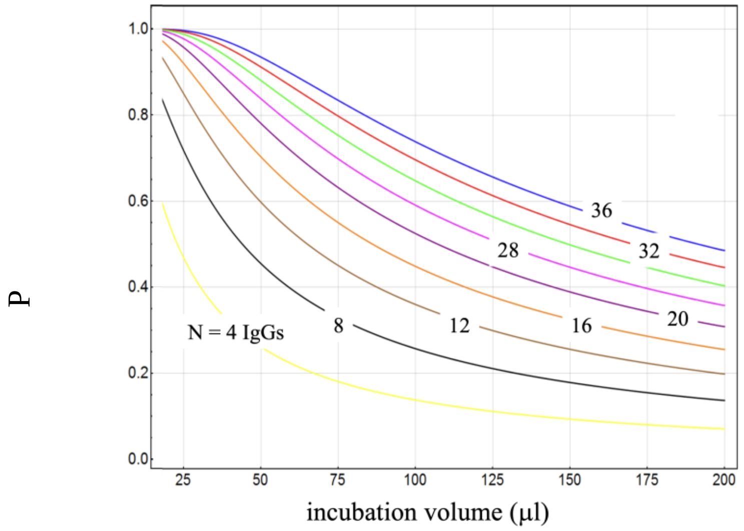

***Figure S4** - Probability P that in an incubation time of 600 s at least one of N IgGs, with N ranging between 4 and 36, will be close enough to the gate so as, during its random Brownian motion, the binding with an anti-IgG will occur. The plot starts from 16 μl, corresponding to the minimum volume needed to cover the gate.*

Table S1: values for all the parameters present in **P**

Physical parameter	***description***	***value/origin***
r_g	Gate radius<br>*Measured*	0.25 cm
Δt	incubation time<br>*Measured*	30 s, 1 min, 5 min, 10 min and 20 min
N	number of particles in the assayed volume V<br>N = [C] (V·N_A) with N_A = Avogadro's number and	from 4 ± 2 to 3.92 $10^7 \pm 6 \cdot 10^3$

	[C] = molar concentration<br>*Measured*	
V	assayed incubation volume<br>*Measured*	25 μl, 100 μl, 1 ml
T	Temperature<br>*Measured*	24 °C
K_B	Boltzmann constant	$1.38064852 \cdot 10^{-23}$ m^2 kg s^{-2} K^{-1}
D	Diffusion coefficient of IgG monomer (T. Jøssang, J. Feder, E. Rosenqvist, J. Protein Chem. **1988**, 7, 165)<br>*Measured*	$3.89 \cdot 10^{-7}$ cm^2/s

Section 6: Chi-squared test

The chi-squared (χ^2) test obtained by fitting experimental data in **Figure 2** with **the probability function P**, by considering just the first experimental point for the saturation plateau (k = degree of freedom; p = probability > χ^2)[8], are:

V = 25 μl, χ^2 = 0.073 (k = 2), p = 0.96;

V = 100 μl, χ^2 = 0.051 (k = 2), p = 0.97;

V =   1 ml, χ^2 = 0.093 (k = 2), p = 0.955;

The chi-squared test for the data in **Figure 3** modelled also with **function P** gives:

χ^2 = 0.0345 (k = 1), p = 0.85.

Since $p > 0.95$ in all the four sets of data at different incubation volumes, while p is larger than 0.85 when fitting the response at different incubation times, there is a high chance (95% for the data of **Figure 2** and 85% for the data of **Figure 3**) that the null hypothesis is correct, *i.e.,* that there is no difference between the observed and theoretical (expected) values predicted by the modeling.

Section 7: Surface Plasmon Resonance study of IgG binding to anti-IgG

The Surface Plasmon Resonance (SPR) technique has been used to characterize the gold surface modified with the bio-recognition element, anti-IgG, in terms of number of antibodies immobilized and the capturing efficacy against the IgG affinity ligand. A Multi-Parameter SPR (MP-SPR) Navi 200-L apparatus in the Kretschmann configuration was used. An Au coated (~ 50 nm) SPR slides (BioNavis Ltd) comprising a chromium adhesion layer (~2 nm) served as semi-transparent SPR substrate. The SPR slide holding an area of 0.42 cm^2 was inspected in two different spots by two laser sources, both set at 670 nm, to estimate the layer homogeneity. The SPR slide was cleaned in a NH_4OH/ H_2O_2 aqueous solution (1:1:5 v/v) at 80-90°C for 10 min, and treated in an ozone cleaner for 10 min. The gold surface was modified *ex-situ* with a mixed self-assembled monolayer (SAM) of alkylthiols: 11-mercaptoundecanoic acid (11MUA) and 3-mercaptopropionic acid (3MPA) in molar ratio 1:10 in ethanol holding a concentration of 10 mM. The sample, immersed in the thiol solution, was left overnight in nitrogen atmosphere at room temperature. Afterwards, the slide was rinsed in ethanol and mounted in the SPR sample holder. The modified SPR slide was further bio-functionalized in the SPR apparatus, thus the real-time anchoring of antibodies on the SAM was monitored *in-situ* as depicted in **Figure S5a**. To achieve the bio-conjugation of antibodies on the SAM, the established coupling method with 1-Ethyl-3-(3-dimethylamino-propyl)carbodiimide (EDC) and N-hydroxysulfosuccinimide sodium salt (NHSS) was used. To this aim, the SAM surface was kept in contact with the aqueous solution of EDC/NHSS (0.2/0.05 M) for 20 minutes. The carboxylic terminal groups of the chemical SAM are converted into intermediate reactive species (NHSS, N-hydroxysulfosuccinimide esters), reacting with the amine groups of the antibody, anti-IgG, achieving its covalent coupling. This was carried out by static injection of 100 µL of the phosphate buffer solution (PBS, ionic strength i_s 163 mM and pH 7.4) of the anti-IgG capturing antibodies (100 µg/mL) in the SPR cell. Then, the ethanolamine saturated solution (EA, at concentration 1 M) is injected to deactivate the unreacted esters in an inactive hydroxyethyl amide. Finally, to cover possible

voids on the SAM and to prevent non-specific binding, a BSA solution 100 μg/mL in PBS was used.

The surface coverage of anti-IgG covalently bound on the SAM was quantitatively assed by means the de Feijter's equation (**Equation S6.1**):

$$\Gamma = d \cdot (n - n_0) \cdot ({}^{dn}/_{dC})^{-1} \qquad \text{(S6.1)}$$

where Γ, expressed in ng cm^{-2}, is the surface coverage, d the thickness of the biolayer deposited on the gold surface, n-n_0 is the difference between the refractive index of the adlayer and the one of the bulk medium, and dn/dC is the specific refractivity of the adsorbed biolayer. Deriving this further to consider the instrument response, it returns **Equation S6.2**:

$$(n - n_0) = \Delta\theta_{SPR} \cdot k \qquad \text{(S6.2)}$$

where k is the wavelength dependent sensitivity coefficient, and $\Delta\theta_{SPR}$ is the experimental angular shift. For laser beams with λ = 670 nm and a thin layers (d < 100 nm), the following approximations hold true: (i) dn/dC ≈ 0.182 cm^3 g^{-1}, (ii) k·d ≈ 1.0·10^{-7} cm deg.[9] Therefore, under these assumptions and by substitution of **Equation S6.1** in **Equation S6.2**, **Equation S6.3** is derived to estimate the surface coverage Γ using the experimental angular shift, being

$$\Gamma = \Delta\theta_{SPR} \cdot 550\ [ng/cm^2] \qquad \text{(S6.3).}$$

Thus, in **Figure S5b** (red squares) the dose-curve for the assayed IgG is reported as $\Delta\theta_{SPR}$ *vs.* [IgG] nominal concentrations (semi-log scale). A control experiment was performed by using the BSA instead of anti-IgG as capturing bio-recognition element. Therefore, the SAM was modified with a BSA solution in PBS at concentration 100 μg/mL, following the same protocol previously described for the anti-IgG anchoring.

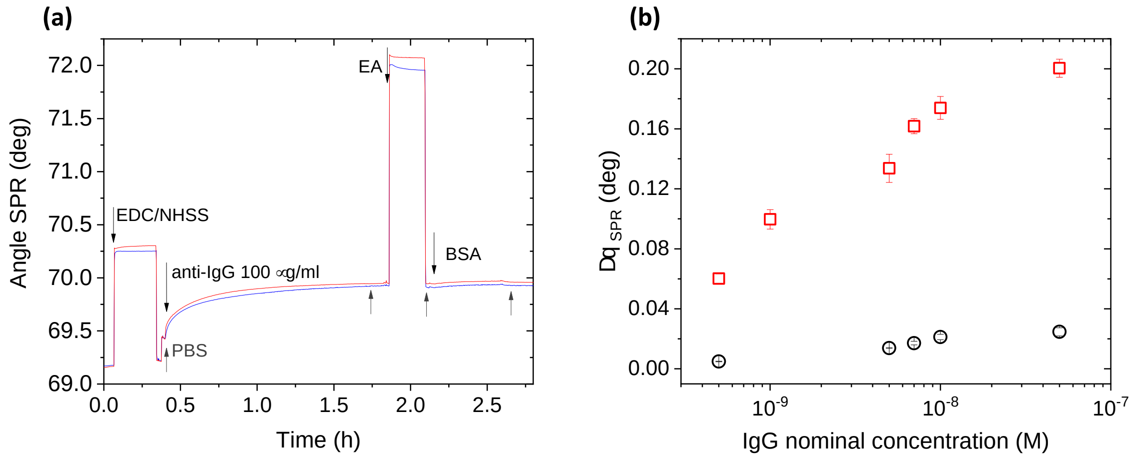

Figure S5 *–* ***(a)*** *SPR sensogram (plasmon peak angle vs. time) of the anti-IgG covalent immobilization through mixed-SAM on the gold SPR slide. Red and blue curves are relevant to two points of the slide surface simultaneously inspected.* ***(b)*** *SPR dose-curves ($\Delta\theta_{SPR}$ vs. [IgG] nominal concentrations on semi-log scale) for the IgG binding on anti-IgG (red squares) and on BSA (black circles) covalently bound on the chemical SAM.*

In **Figure S5b** (black circles) the response of the control sample upon the exposure of IgG is reported as well as $\Delta\theta_{SPR}$ *vs.* [IgG] nominal concentrations. An anti-IgG surface coverage of 288 ± 17 ng cm^{-2}, corresponding to (1.16 ± 0.3) $\cdot 10^{12}$ molecules$\cdot$cm^{-2}, has been registered 1 hour and 30 minutes after the PBS washing step. The binding efficacy of the bio-recognition elements immobilized on the SAM was tested against IgG. The analysis was carried out by recording the baseline in PBS and injecting IgG solutions in PBS at different concentrations, ranging from $5\cdot10^{-10}$ M to $5\cdot10^{-8}$ M. The angular shift, $\Delta\theta_{SPR}$, was calculated for each concentration as the difference between the equilibrium value, after rinsing with PBS, and the initial baseline.

References

[1] A. I. ElSherbini, A. M. Jacobi, *J. Colloid Interface Sci.* **2004**, *273*, 556.

[2] H. Elwing, *FEBS Lett.* **1980**, *116*, 239.

[3] R. D. Deegan, O. Bakajin, T. F. Dupont, G. Huber, S. R. Nagel, T. A. Witten, *Nature* **1997**, *389*, 827.

[4] R. G. Larson, *In Retrospect: Twenty years of drying droplets*, Vol. 550, **2017**, pp. 466–467.

[5] D. S. Viswamath, T. Ghodh, D. H. L. Prasad, N. V. K. Dutt, K. Y. Rani, *Single comprehensive book on viscosity of liquids Gathers the description and analysis of the experimental methods and governing theory are not readily available in a single volume*, Vol. 49, **2007**.

[6] T. Jøssang, J. Feder, E. Rosenqvist, *J. Protein Chem.* **1988**, *7*, 165.

[7] D. L. Ermak, J. A. McCammon, *J. Chem. Phys.* **1978**, *69*, 1352.
[8] K. Larntz, *J. Am. Stat. Assoc.* **1978**, *73*, 253.
[9] V. Ball, J. J. Ramsden, *Biopolymers* **1998**, *46*, 489.
